# Evaluation of Endoscopic Ultrasound Delays in the Diagnosis of Pancreatic Cancer in Older Adults in the United States

**DOI:** 10.14309/ctg.0000000000000986

**Published:** 2026-01-30

**Authors:** Rotana M. Radwan, Wenxi Huang, Grace Barney, Jennifer Fieber, Jingchuan Guo, Aleksey Novikov

**Affiliations:** 1 Department of Pharmaceutical Outcomes and Policy, University of Florida College of Pharmacy, Gainesville, Florida, USA;; 2 Department of Pharmacy Practice, Purdue University College of Pharmacy, Indianapolis, Indiana, USA;; 3 Department of Biology, University of Florida College of Liberal Arts and Sciences, Gainesville, Florida, USA;; 4 Department of Surgery, Division of Surgical Oncology, University of Florida College of Medicine, Gainesville, Florida, USA;; 5 Regenstrief Institute, Indianapolis, Indiana, USA;; 6 Department of Medicine, Division of Gastroenterology, Hepatology, and Nutrition, University of Florida College of Medicine, Gainesville, Florida, USA.

**Keywords:** pancreatic ductal adenocarcinoma, endoscopic ultrasound, mortality risk, metastatic disease, socioeconomic disparities

## Abstract

**INTRODUCTION:**

Pancreatic cancer is among the most aggressive malignancies, with a 5-year survival rate of 10%. Most patients present with advanced disease, limiting curative treatment options. Endoscopic ultrasound with fine-needle biopsy is the standard for diagnosis and staging. Although early access to endoscopic ultrasound (EUS) may enable timely systemic therapy and improve resectability, uncertainty remains regarding how delays to EUS affect surgical resection rates and overall survival, particularly in older adults. We aimed to identify factors associated with delayed EUS and to evaluate its impact on surgical resection and overall survival.

**METHODS:**

Using national Medicare claims (2011–2020), we conducted a retrospective cohort study of beneficiaries aged 66 years or older with newly diagnosed pancreatic cancer. The index date was the most recent claim for a pancreatic lesion or abnormal liver enzymes, serving as the indicator for EUS referral. Delay to EUS was defined as >30 days between the index date and the EUS procedure. Multivariable logistic regression identified sociodemographic and clinical factors associated with delayed EUS. Cox proportional hazards models estimated the associations between delayed EUS and 2 outcomes: (i) pancreatic surgical resection and (ii) all-cause mortality.

**RESULTS:**

Among 2,843 patients, 586 (20.6%) experienced a delay in EUS, 774 (27.2%) underwent surgery, and 1,591 (56.0%) died. Black patients were more likely to experience delay (adjusted odds ratio 1.65, 95%CI 1.09–2.51), whereas those with more comorbidities were less likely (adjusted odds ratio 0.95, 95%CI 0.90–0.99). Delayed EUS was associated with a lower likelihood of surgery (hazard ratio [HR] 0.73, 95%CI 0.61–0.88) but lower mortality (HR 0.58, 95%CI 0.50–0.66). Mortality increased with older age (HR 1.43, 95%CI 1.27–1.61) and comorbidity (HR 1.04, 95%CI 1.02–1.07).

**DISCUSSION:**

Timely EUS was associated with higher surgical resection rates, suggesting earlier access to curative treatment. Lower mortality among patients with delayed EUS possibly reflects disease severity confounding rather than benefit.

## INTRODUCTION

Pancreatic cancer is among the most aggressive cancers, with a 5-year overall survival rate of 10%, and is projected to be the second-leading cause of cancer death in the United States by 2030 (1). Pancreatic cancer is a diagnostic challenge, with 25%–30% of patients presenting with locally advanced disease and over 50% of patients presenting with distant metastatic disease at the time of diagnosis (1,2). Treatment of early and locally advanced pancreatic cancer is often multimodal, consisting of a combination of surgical resection and systemic therapy (2,3).

The role of endoscopic ultrasound (EUS) with fine needle biopsy is becoming increasingly important in the diagnosis of pancreatic cancer (4). EUS provides a definitive diagnosis in over 90% of cases, with low complication rates (5). The EUS procedure can offer additional information about staging and resectability. EUS is also superior to cross-sectional imaging for evaluating small tumors (5,6). Endoscopic management of EUS with endoscopic retrograde cholangiopancreatography and stenting can relieve biliary obstruction, increasing patients' comfort and ability to receive additional treatment (7).

As a minimally invasive procedure, EUS enables early tissue acquisition and supports treatment strategies such as neoadjuvant chemotherapy or targeted therapies aimed at improving resectability of locally advanced disease (7,8). The evolution of targeted therapies and advances in tumor genomics have shown promise for improving pancreatic cancer outcomes in the future, and early tissue diagnosis will make more patients eligible for these modalities (2). EUS has been independently associated with improved survival, curative-intent surgery, and chemoradiation in locally advanced pancreatic cancer (9).

Early-stage disease at presentation and expedient treatment are critical for achieving favorable outcomes in pancreatic cancer. However, the diagnostic pathway from initial presentation to tissue confirmation may introduce delays that could affect disease progression and treatment options. Although EUS represents an essential step to confirm diagnosis, assess resectability, and guide treatment planning, the time from clinical presentation of a pancreatic neoplasm to EUS completion remains poorly characterized, as do the sociodemographic and clinical factors that influence this interval. Furthermore, whether delays in obtaining tissue diagnosis affect surgical eligibility and survival outcomes is not well established. To evaluate these gaps, we performed a retrospective analysis of older adults diagnosed with pancreatic cancer after an EUS procedure to: (i) identify sociodemographic and clinical factors associated with prolonged time to EUS, and (ii) examine the relationship between EUS timing and both surgical resectability and mortality. Understanding diagnostic delays and their determinants is essential for identifying opportunities to expedite care and potentially improve outcomes in this aggressive malignancy.

## METHODS

### Study design, data source, and sample

We conducted a retrospective cohort study using Medicare administrative claims data, which provide comprehensive, longitudinal, individual-level information on demographics, diagnoses, procedures, and pharmacy claims for persons aged 65 years or older in the United States. A random sample of national Medicare claims data from January 1, 2011, to December 31, 2016 (5% samples) and January 1, 2017, to December 31, 2020 (15% samples) was accessed for this study. The study adhered to STROBE reporting guidelines and was deemed exempt from review by the University of Florida Institutional Review Board.

Patient selection was initially based on the presence of diagnostic codes for pancreatic neoplasms, pancreatic inflammation, or abnormal liver enzymes. Pancreatic disorders (*ICD10*; *ICD9*) included acute pancreatitis (K85.90–K85.92; 577.0), chronic pancreatitis (K86.1; 577.1), pancreatic cysts (K86.0; 577.2), steatorrhea (K86.81; 577.2), and other or unspecified pancreatic diseases (K86.8, K86.9; 577.8). Liver-related findings were captured using codes for abnormal imaging (R93.3; 793.19), jaundice (R17; 782.4), ascites (R18; 789.5), and abnormal liver function tests (R94.5; 794.8). These codes were used to ensure consistency across Data sets and to facilitate accurate identification of relevant clinical conditions.

**Figure 1.**
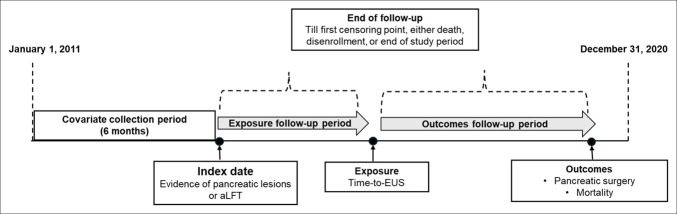
Schematic representation of study design. aLFT, abnormal liver function test; EUS, endoscopic ultrasound.

Eligible patients were required to have at least 6 months of continuous enrollment in Medicare fee-for-service (Parts A, B, and D) before the index date and maintain coverage throughout the follow-up period. Patients were excluded if they had a documented pancreatic cancer diagnosis (C25. x; 157.x) *before* the index date or did not undergo an EUS procedure after the identification of pancreatic lesions or elevated liver function tests (LFTs). The index date was defined as the most recent claims-based record of a pancreatic lesion or elevated LFTs before the EUS. Because we required at least 6 months of previous Medicare enrollment, the final study cohort consisted of beneficiaries aged 66 years or older who were diagnosed with a malignant neoplasm of the pancreas after undergoing an EUS procedure during the study period (Figure 1).

### Exposure and outcomes

The primary variable of interest was time-to-EUS, defined as the number of days between the first claims-based indication of a pancreatic lesion or elevated LFTs and the date of the EUS performed to evaluate that lesion. The occurrence of EUS was defined by Current Procedural Terminology (CPT) codes 43248 and 43242, which indicate endoscopic ultrasound with biopsy. Diagnosis of pancreatic cancer was defined by *ICD-10* and *ICD-9* codes of C25. x and 157.x. Occurrence of pancreatic surgery was defined by distal pancreatectomy CPT codes 48140, 48145, pancreaticoduodenectomy CPT codes 48150, 48152, 48153, 48154, total pancreatectomy CPT code 48155, or near-total pancreatectomy CPT code 48146 (Figure 1).

An interval >30 days was defined as a delay to EUS. The 30-day threshold was chosen for its clinical relevance, because it aligns with established expectations for timely diagnostic evaluation in oncology. Many previous studies on pancreatic cancer and other malignancies have used a 30-day interval to define delay in care (10–13). There is no standardized guideline for defining delays in the care of patients with pancreatic cancer.

Time-to-EUS was analyzed as an outcome to evaluate associations between patient sociodemographic and clinical characteristics and the likelihood of experiencing a delay to EUS. Time-to-EUS was then examined as the main exposure to assess its relationship with downstream outcomes. From the date of EUS, patients were followed for 2 outcomes: (i) pancreatic surgery and (ii) all-cause mortality. Follow-up continued until the earliest of death, Medicare disenrollment, or the end of the study period. A schematic representation of the study design is provided in Figure 1. All codes used to define exposures and outcomes were identified using the International Classification of Diseases, Ninth and Tenth Revisions (*ICD-9* and *ICD-10*), and CPT coding systems.

### Covariates

To account for potential confounding, both sociodemographic and clinical characteristics were included as covariates. Sociodemographic variables comprised age group (≥75 vs <75), sex (male, female), and race/ethnicity categorized as non-Hispanic White, non-Hispanic Black, Hispanic, or Other (including Asian/Pacific Islander, American Indian/Alaska Native, or other). Geographic classification as rural or urban was based on the U.S. Department of Agriculture's 2013 Rural-Urban Continuum Codes, with codes 1–3 defined as urban and codes 4–9 as rural. Region of residence was categorized into 5 U.S. Census divisions: West, Midwest, Northeast, Southwest, and Southeast. Socioeconomic status was assessed using the Area Deprivation Index (ADI), a composite measure of neighborhood disadvantage that incorporates 17 U.S. Census variables (e.g., education, income, employment, housing, and transportation access). For analysis, the national ADI percentile ranking was used (range: 1–100), with higher values indicating greater socioeconomic disadvantage (14,15).

Clinical characteristics included the Charlson Comorbidity Index (CCI), a validated scoring system that quantifies overall comorbidity burden based on the presence of 17 chronic conditions predictive of 1-year mortality. Each condition is assigned a weight (ranging from 1 to 6), and the total score reflects the cumulative burden of illness. Higher CCI scores indicate more severe comorbidity and are associated with an increased risk of 1-year mortality (16). In this study, the CCI was treated as a continuous variable (17).

### Statistical analysis

Descriptive statistics were used to summarize baseline patient characteristics overall and stratified by time-to-EUS (≤30 vs >30 days). Continuous variables were reported as means with SDs or medians with IQRs, as appropriate. Categorical variables were summarized as counts and percentages. Group comparisons were conducted using the χ^2^ test for categorical variables, and either the 2-sample *t*-test or the Wilcoxon rank-sum test for continuous variables.

To identify clinical and sociodemographic factors associated with diagnostic delay, we fit a multivariable logistic regression model with time-to-EUS as the binary dependent variable (≤30 days; >30 days). Adjusted odds ratios (aORs) with 95% CIs were estimated.

Time-to-EUS was then treated as the primary exposure to assess whether the duration of diagnostic delay influenced clinical outcomes. Specifically, 2 separate multivariable Cox proportional hazards models were used to evaluate the association between time-to-EUS (categorized as ≤30 vs >30 days) and the risk of (i) undergoing pancreatic surgery and (ii) all-cause mortality, with follow-up beginning at the date of the EUS procedure. Each model estimated adjusted hazard ratios (HRs) with corresponding 95% CIs.

To assess the robustness of the findings across alternative definitions of diagnostic delay, sensitivity analyses were performed using different time-to-EUS thresholds. These included an 8-day cutoff (corresponding to the median time to EUS in the cohort) to represent a more stringent definition of delay and a 60-day cutoff to represent a more permissive definition.

All models were adjusted for baseline covariates to account for differences in patient characteristics and to reduce potential confounding. Statistical analyses were performed using SAS version 9.4 (SAS Institute, Cary, NC). Two-sided *P*-values <0.05 were considered statistically significant.

## RESULTS

### Study population

**Figure 2.**
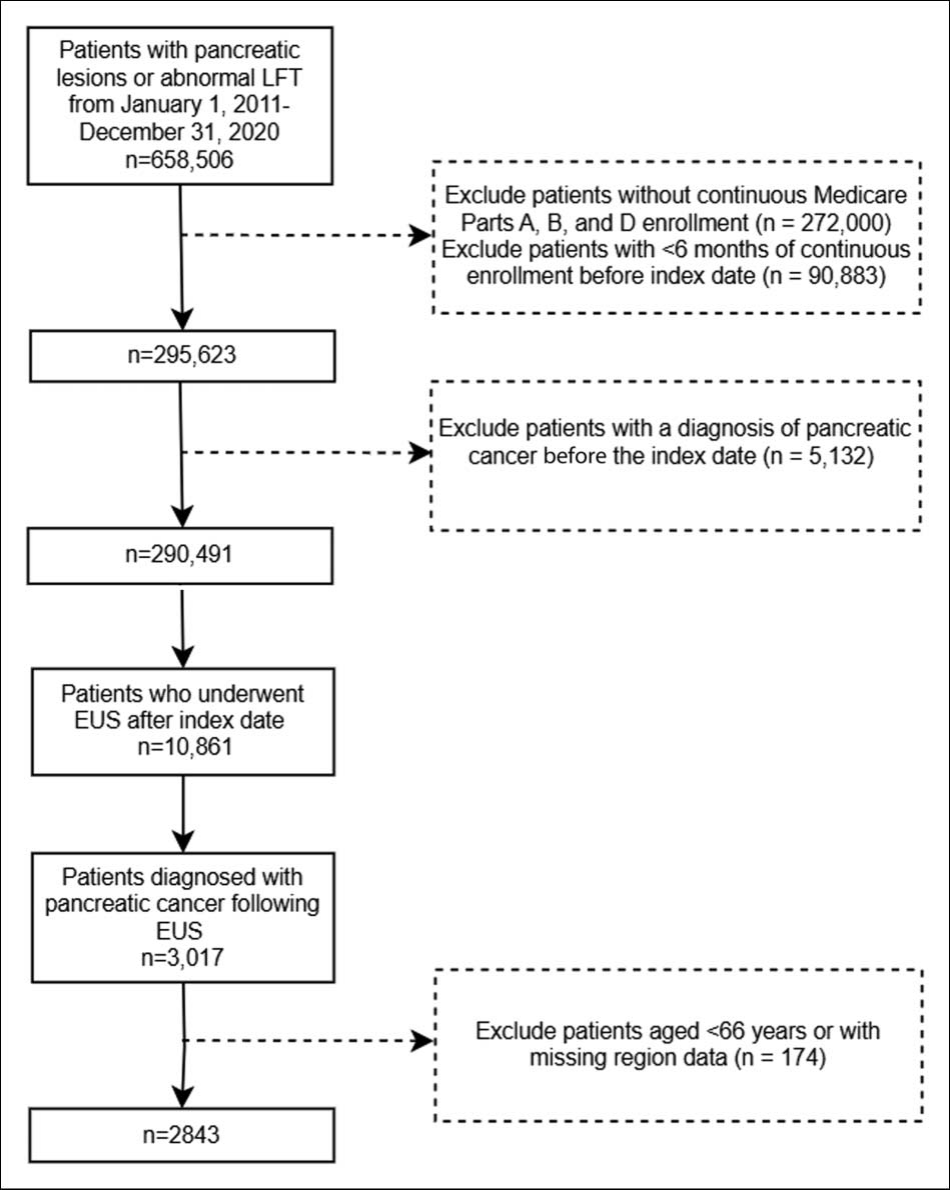
Patient attrition flow chart. EUS, endoscopic ultrasound; LFT, liver function test.

Initially, 658,506 patients with pancreatic lesions or elevated LFTs were identified in our analysis. After exclusion of people with less than 6 months of continuous Medicare A, B, and D coverage and patients with a pancreatic cancer diagnosis before the study period, 290,491 patients were identified. Of those, 10,861 patients underwent an EUS, with 3,017 diagnosed with pancreatic cancer after the EUS study. An additional 174 patients were excluded due to missing data, leaving a final study cohort of 2,843 patients (Figure 2).

The cohort was predominantly non-Hispanic White (88.6%), aged 75 years or older (51.6%), and men (51.1%). Most patients resided in the Southeast (44.1%) and in urban areas (85.1%). The mean national rank for the ADI was 43.9 (SD = 21.8), indicating a middle-range level of neighborhood socioeconomic disadvantage on the national 1–100 scale. The median CCI was 2.00 (IQR: 1.00–4.00). Overall, baseline characteristics were similar between patients with and without diagnostic delays (Table 1).

**Table 1.** Overall and stratified characteristics of patients with pancreatic cancer who underwent EUS

Variable	Overall cohort (n = 2,843)	Delay to EUS (n = 585; 20.57%)	No delay (n = 2,258; 79.43%)	*P*-value
Age, n (%)				
75+	1,468 (51.64%)	291 (49.74%)	1,177 (52.13%)	0.304
<75	1,375 (48.36%)	294 (50.26%)	1,081 (47.87%)	
Sex, n (%)				
Male	1,452 (51.07%)	296 (50.60%)	1,156 (51.20%)	0.797
Female	1,391 (48.93%)	289 (49.40%)	1,102 (48.80%)	
Race, n (%)				
Non-Hispanic White	2,518 (88.57%)	502 (85.81%)	2016 (89.28%)	0.071
Non-Hispanic Black	128 (4.50%)	37 (6.32%)	91 (4.03%)	
Hispanics	79 (2.78%)	18 (3.08%)	61 (2.70%)	
Other	118 (4.15%)	28 (4.79%)	90 (3.99%)	
Region, n (%)				
West	321 (11.29%)	73 (12.48%)	248 (10.98%)	0.497
Midwest	549 (19.31%)	101 (17.26%)	448 (19.84%)	
Northeast	528 (18.57%)	103 (17.61%)	425 (18.82%)	
Southwest	190 (6.68%)	40 (6.84%)	150 (6.64%)	
Southeast	1,255 (44.14%)	268 (45.81%)	987 (43.71%)	
Residence location, n (%)				
Rural	2,420 (85.12%)	503 (85.98%)	1917 (84.90%)	0.511
Urban	423 (14.88%)	82 (14.02%)	341 (15.10%)	
Area Deprivation Index, mean (SD)	43.92 (21.83)	45.44 (22.05)	43.53 (21.77)	0.062
Charlson Comorbidity Index, median (IQR)	2.00 (1.00, 4.00)	2.00 (1.00, 4.00)	2.00 (1.00, 4.00)	0.414

EUS, endoscopic ultrasound.

### Time to EUS

Of the 2,843 diagnosed with pancreatic cancer after EUS, 20.6% had a time from onset of concern to EUS greater than 30 days (termed delay to EUS), and 79.4% underwent EUS within 30 days. The mean time to EUS was 42.0 days (SD: 135.0), and the median time was 8 days (IQR: 1–24).

### Factors associated with time-to-EUS

We were interested in sociodemographic and clinical factors associated with a delay to EUS. We performed a multivariable logistic regression analysis examining various covariates. Compared with non-Hispanic White individuals, non-Hispanic Black individuals had higher odds of experiencing a delay (aOR = 1.65; 95% CI: 1.09–2.51; *P* = 0.019). By contrast, greater comorbidity burden, as measured by CCI, was associated with lower odds of delay (aOR = 0.95; 95% CI: 0.90–0.99; *P* = 0.017). There was no significant association with ADI, age, sex, region, or residence location (Table 2).

**Table 2.** Multivariable logistic regression results of factors associated with diagnostic delay

Variable	aOR	95% CI	*P*-value
Area Deprivation Index	1.01	1.00–1.01	0.062
Charlson Comorbidity Index	0.95	0.90–0.99	0.017
Age (ref: <75 yr)			
≥75 yr	0.83	0.67–1.04	0.103
Sex (ref: female)			
Male	0.93	0.72–1.22	0.618
Race/Ethnicity (ref: White)			
Black	1.65	1.09–2.51	0.019
Hispanic	1.19	0.68–2.09	0.538
Other	1.28	0.81–2.01	0.291
Region (ref: Southeast)			
West	1.22	0.88–1.68	0.240
Midwest	0.85	0.65–1.12	0.247
Northeast	1.05	0.80–1.38	0.733
Southwest	0.98	0.67–1.44	0.909
Residence location (ref: rural)			
Urban	1.16	0.86–1.56	0.335

aOR, adjusted odds ratio.

Note: Additional covariates were adjusted in the model including: acute myocardial infarction, the Alzheimer disease, atrial fibrillation, cataract, chronic kidney disease, chronic obstructive pulmonary disease, congestive heart failure, diabetes, glaucoma, ischemic heart disease, depression, osteoporosis, rheumatoid arthritis and/or osteoarthritis, stroke and/or transient ischemic attack, anemia, asthma, hyperlipidemia, hyperparathyroidism, hypertension, hypothyroidism, alcohol use disorder, and obesity.

### Association between time-to-EUS and undergoing pancreatic surgery

**Figure 3.**
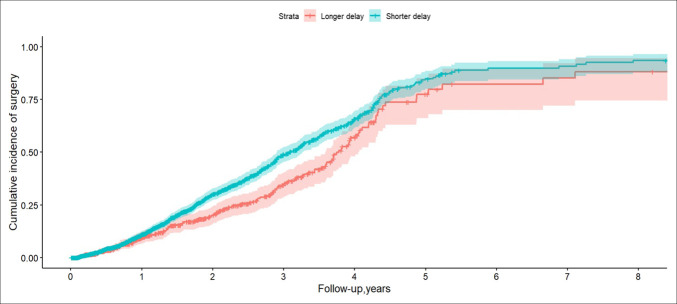
Kaplan-Meier curves depicting the cumulative incidence of surgery among patients with pancreatic cancer, stratified by diagnostic delay group. The figure shows the cumulative incidence of surgery over an 8-year follow-up period for patients with shorter (<30 days) versus longer (>30 days) diagnostic delays. The upper curve represents patients with a shorter diagnostic delay, whereas the lower curve represents those with a longer diagnostic delay. Shaded areas indicate 95% CIs. Patients with shorter diagnostic delays had higher cumulative incidence of undergoing surgery.

Of the 2,843 patients in the sample, diagnosed with pancreatic cancer, 774 (27.2%) underwent pancreas surgery during the follow-up period. The median time from EUS to surgery was 46.5 days (IQR: 26–133), and the mean time was 95.4 days (SD: 124.2). In the multivariable Cox regression analysis, a delay to EUS was associated with a significantly lower likelihood of undergoing pancreatic surgery compared with patients who had EUS within 30 days of diagnosis of pancreatic neoplasm (HR = 0.73; 95% CI: 0.61–0.88; *P* < 0.001) (Figure 3). Residence in the Northeast was also associated with a lower likelihood of undergoing pancreatic surgery compared with residence in the Southeast (HR = 0.79; 95% CI: 0.64–0.97; *P* = 0.026). Patients with a higher CCI score (HR = 0.95; 95% CI: 0.91–0.99; *P* = 0.017) were less likely to undergo pancreas surgery. Age, sex, race, and residence were not significantly associated with changes in surgery rates (Table 3).

**Table 3.** Multivariable cox proportional hazards model of time to pancreatic surgery

Variable	HR	95% CI	*P*-value
Time-to-EUS (ref: ≤30 d)			
>30 d	0.73	0.61–0.88	0.001
Area Deprivation Index	1.00	0.99–1.00	0.108
Charlson Comorbidity Index	0.95	0.91–0.99	0.017
Age (ref: <75 yr)			
≥75 yr	1.09	0.92–1.29	0.340
Sex (ref: female)			
Male	0.86	0.71–1.05	0.144
Race/Ethnicity (ref: White)			
Black	0.69	0.45–1.07	0.101
Hispanic	1.34	0.89–2.02	0.164
Other	0.98	0.70–1.36	0.895
Region (ref: Southeast)			
West	0.89	0.69–1.15	0.383
Midwest	0.86	0.70–1.06	0.154
Northeast	0.79	0.64–0.97	0.026
Southwest	0.80	0.58–1.10	0.168
Residence location (ref: rural)			
Urban	0.84	0.66–1.06	0.135

EUS, endoscopic ultrasound; HR, hazard ratio.

Note: Additional covariates were adjusted in the model including: acute myocardial infarction, the Alzheimer disease, atrial fibrillation, cataract, chronic kidney disease, chronic obstructive pulmonary disease, congestive heart failure, diabetes, glaucoma, ischemic heart disease, depression, osteoporosis, rheumatoid arthritis and/or osteoarthritis, stroke and/or transient ischemic attack, anemia, asthma, hyperlipidemia, hyperparathyroidism, hypertension, hypothyroidism, alcohol use disorder, and obesity.

### Association between time-to-EUS and all-cause mortality

**Figure 4.**
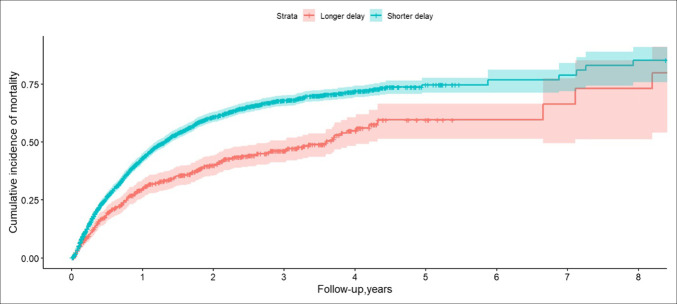
Kaplan-Meier curves depicting the cumulative incidence of mortality among patients with pancreatic cancer, stratified by diagnostic delay. The figure displays the cumulative incidence of mortality over an 8-year follow-up period for patients with shorter (<30 days) versus longer (>30 days) diagnostic delays. The upper curve represents patients with a shorter diagnostic delay, and the lower curve represents those with a longer diagnostic delay. Shaded areas indicate 95% CIs. Patients with shorter diagnostic delays had a higher cumulative incidence of mortality.

Of the 2,843 patients who were diagnosed with pancreatic cancer, 1,591 (56.0%) died during the follow-up period. The median time from EUS to death was 264 days (IQR: 114–487), and the mean was 359.9 days (SD: 352.2). In the multivariable Cox regression analysis for all-cause mortality, several factors were significantly associated with differences in risk. Interestingly, patients who had a delay to EUS had a significantly lower risk of mortality (HR = 0.58; 95% CI: 0.50–0.66; *P* < 0.001) (Figure 4). Patients aged 75 and older (HR = 1.43; 95% CI: 1.27–1.61; *P* < 0.001) and with higher CCI (HR = 1.04; 95% CI: 1.02–1.07; *P* = 0.001) had a higher likelihood of all-cause mortality. Rural residence was associated with a lower risk of death compared with urban residence (HR = 0.85; 95% CI: 0.73–0.99; *P* = 0.033) (Table 4).

**Table 4.** Multivariable cox regression results for all-cause mortality

Variable	HR	95% CI	*P*-value
Time to EUS (ref: <30 d)			
>30 d	0.58	0.50–0.66	<.0001
Area Deprivation Index	1.002	0.00–1.01	0.139
Charlson Comorbidity Index	1.04	1.02–1.07	0.001
Age (ref: <75 yr)			
75+	1.43	1.27–1.61	<.0001
Sex (ref: Female)			
Male	0.99	0.00–1.14	0.892
Race/Ethnicity (ref: White)			
Black	1.10	0.00–1.41	0.444
Hispanic	0.95	0.00–1.32	0.765
Other	0.78	0.00–1.04	0.092
Region (ref: Southeast)			
West	1.05	0.00–1.25	0.632
Midwest	1.03	0.00–1.18	0.728
Northeast	1.05	0.00–1.21	0.556
Southwest	0.97	0.00–1.19	0.751
Residence location (ref: Rural)			
Urban	0.85	0.73–0.99	0.033

EUS, endoscopic ultrasound; HR, hazard ratio.

Note: Additional covariates were adjusted in the model, including: acute myocardial infarction, the Alzheimer disease, atrial fibrillation, cataract, chronic kidney disease, chronic obstructive pulmonary disease, congestive heart failure, diabetes, glaucoma, ischemic heart disease, depression, osteoporosis, rheumatoid arthritis and/or osteoarthritis, stroke and/or transient ischemic attack, anemia, asthma, hyperlipidemia, hyperparathyroidism, hypertension, hypothyroidism, alcohol use disorder, and obesity.

### Sensitivity analyses

To assess the robustness of our primary findings, we performed sensitivity analyses using alternate thresholds to define delay (8 and 60 days). In the logistic regression models, higher comorbidity burden was protective against delay at the 8-day threshold (aOR = 0.94; 95% CI: 0.91–0.98; *P* = 0.001), whereas older age (≥75 years) was protective at the 60-day threshold (aOR = 0.72; 95% CI: 0.54–0.95; *P* = 0.020). The association between delay and reduced likelihood of surgery remained consistent across all thresholds (8-day: HR = 0.86; 95% CI: 0.74–1.00; *P* = 0.043; 60-day: HR = 0.73; 95% CI: 0.57–0.92; *P* = 0.009). Similarly, the association between delay and lower all-cause mortality persisted across thresholds (8-day: HR = 0.82; 95% CI: 0.74–0.91; *P* < 0.001; 60-day: HR = 0.62; 95% CI: 0.51–0.74; *P* < 0.001). Detailed results are presented in Supplementary Tables 1–6 (see Supplementary Digital Content, http://links.lww.com/CTG/B464).

## DISCUSSION

To our knowledge, this is the first study to assess the timing of EUS in older adults with pancreatic cancer and its association with clinical outcomes. In this national cohort of 2,843 patients, we found that approximately 1 in 5 patients (20.6%) experienced a delay of more than 30 days from initial concerning findings to EUS. Our key findings include: (i) Black patients were significantly more likely to experience delayed EUS, even after adjusting for clinical and sociodemographic factors; (ii) patients who underwent EUS within 30 days were significantly more likely to undergo surgical resection; and (iii) paradoxically, delayed EUS was associated with lower all-cause mortality.

Our finding that Black patients were significantly more likely to experience delayed EUS (aOR 1.65, 95% CI 1.09–2.51) adds to a growing body of evidence documenting racial disparities across the pancreatic cancer care continuum. Previous studies, including Silva-Santisteban et al, have demonstrated multifactorial disparities in access to pancreatic cancer screening, particularly EUS (18). Although geographic distance from EUS-trained endoscopists has been associated with delayed care and mortality in esophageal cancer (19), this association has not been confirmed in pancreatic cancer. Our results align with those of Fallon et al, who reported delayed biopsy and treatment among underrepresented minorities with pancreatic cancer (20). Black patients are more likely to develop pancreatic cancer, present at later stages, and experience worse outcomes (21,22). Even at the same stage, they are less likely to receive oncology consultations, surgery, or chemotherapy (21,23). The Black race has also been identified as a risk factor for treatment delay (24). Interestingly, in our study, patients with higher CCI scores were slightly less likely to have EUS delays, possibly reflecting more frequent healthcare encounters and closer clinical monitoring.

In our cohort, 27.2% of patients underwent surgical resection of pancreatic cancer. Patients who underwent EUS within 30 days were significantly more likely to undergo surgery. Pancreatic resection serves as a proxy for earlier-stage disease, as surgical management is only an option for early or locally advanced disease that was deemed resectable either before or after neoadjuvant treatment. This result has 2 potential explanations. First, it may suggest that patients who are thought to be potentially curable may be prioritized for the EUS procedure either to expedite surgical management or start systemic therapy to bridge to resection over patients with more advanced, metastatic disease. Alternatively, patients who had earlier EUS may have been less likely to experience delays in care that could affect candidacy for resection. Our study also found that patients with higher CCI, were less likely to undergo resection. CCI can be viewed as a proxy for surgical candidacy, and patients with higher CCI are likely poorer surgical candidates and are less likely to be offered a surgical intervention.

Despite patients with a delay to EUS being less likely to undergo surgical resection, all-cause mortality was lower in this group. This pattern may seem counterintuitive. Medicare claims do not provide cancer-specific mortality or detailed clinical factors such as tumor stage, tumor burden, functional status, symptom severity, or laboratory values. The association between delayed EUS and lower all-cause mortality should be interpreted with caution and may reflect residual confounding rather than some protective effect of delay. One explanation is that patients undergoing earlier EUS may represent those with greater clinical urgency, whereas patients with longer time-to-EUS may be more clinically stable and/or have less aggressive disease at presentation. On the other hand, as the use of neoadjuvant chemotherapy and next-generation sequencing after a tissue diagnosis becomes more common, it is possible that patients who experience a delay in EUS due to a more advanced disease will receive neoadjuvant chemotherapy and possibly radiation before ever undergoing surgery, which may explain our findings of improved mortality in patients with a delay to EUS. This was a study conducted among older patients with pancreatic cancer who may choose against surgery in a setting of more advanced disease. In addition, the claims-based index date may not perfectly capture the true timing of the initial abnormal finding, potentially leading to misclassification of the time-to-EUS interval. Sensitivity analyses using alternative delay thresholds (>8 and >60 days) yielded consistent findings, supporting robustness but not establishing causality. Overall mortality in our study was very high at 56.0%, as expected in older patients with pancreatic cancer and increased comorbidity burden, as reflected by the CCI.

Delays in treatment have been linked to poorer outcomes in lung, colorectal, bladder, and breast cancers (12). However, unlike many other malignancies, there are currently no established guidelines for pancreatic cancer that define acceptable timeframes from presentation through diagnosis to treatment initiation. This gap is notable given the aggressive biology of pancreatic cancer, with an estimated progression time from T1 to T4 of approximately 14 months and metastatic liver lesions developing in just 3.5 months for tumors larger than 2.5 cm (25–27). These findings suggest that timely workup and initiation of treatment could influence outcomes. However, demonstrating survival differences in pancreatic cancer is challenging due to the overall poor prognosis and the need for large sample sizes to detect meaningful effects. Numerous studies have assessed delays in pancreatic cancer care and outcomes, yielding mixed results.

The literature also lacks consistency in defining the phases of care used to determine delays in care intervals. Time from onset of symptoms to diagnosis is 1 variable in the chain of events needed to treat pancreatic cancer. Our study is unique because it evaluated the time from the first documented concerning finding to the EUS procedure for a definitive diagnosis in a national administrative Data set. Previous research has analyzed intervals from symptom onset, presentation to a physician, first imaging suggestive of a pancreatic mass, confirmed tissue diagnosis, referral placement, visit with a surgeon or consultant, and initiation of treatment of pancreatic cancer (28). Stornello et al published a large case series from Italy showing that the mean or median time to presentation was 2 months (IQR 1–5 months) (29). In a British prospective cohort study (SYMPTOM), the average time to diagnosis from symptom onset was 117 days (95% CI: 95–237) (30). In both studies, there was no survival or staging benefit associated with earlier presentation; however, in both cases, patients took months to present. Another American study sought to explore the association between the time to surgery after EUS biopsy and found that additional EUS biopsies delayed treatment initiation. This study did not assess downstream treatment, survival, or staging outcomes (24). Another study on the delayed treatment of hepatobiliary malignancies found that patients with resectable tumors who had delays in care were less likely to survive (11). Both Gobbie et al and Raptis et al found an association between delays in care and worse survival (31,32). Most recently, de Leon Pisani et al evaluated diagnostic delays and time to treatment in a prospective single institution cohort of 282 patients and found that delays of >104 days from patient-reported symptom onset to first treatment were associated with a higher risk of death (28).

Although there are varying definitions of pancreas care delays and the evaluated time intervals, much of the literature suggests that longer wait times are associated with worse outcomes. In a systematic literature review, Lukács et al reviewed 19 studies and 8 multivariate analyses and found that 5 studies indicated significantly better survival with fewer delays in care (33). Sanjeevi et al evaluated patients with potentially curable pancreatic cancer regarding the time between imaging and treatment and discovered that surgery within 32 days had the highest chance of identifying resectable disease at the time of the operation (34). Deshwar et al conducted a retrospective chart review of 116 patients with pancreatic cancer. They found that patients with less than a 60-day interval from initial presentation to a physician to a confirmed diagnosis of pancreatic cancer were more likely to undergo upfront surgery (25). Our study suggests that EUS within 30 days of symptom onset may be beneficial.

This study has several limitations. As a retrospective observational analysis, the findings are hypothesis-generating and require validation in prospective studies that can better capture disease stage, treatments, and clinical outcomes. Our study involved only patients who underwent EUS and was primarily concerned with the timing of EUS utilization and its effect on surgical outcomes. We did not assess patients diagnosed and surgically treated without EUS, including those with metastatic disease, early-stage cancers undergoing upfront surgery, or those who had tissue sampling by interventional radiology. As mentioned previously, due to the nature of Medicare claims data, we lacked information on key clinical factors, including stage at diagnosis, imaging findings, treatment details, and cancer-specific mortality. We emphasize that oncologic outcomes, such as overall mortality reported in this study, should be interpreted with caution due to several biases and confounders. The absence of a single ICD code for suspected malignant pancreatic lesions necessitated the use of multiple diagnostic codes, which may have introduced misclassification. In addition, coding completeness may affect data accuracy, and our definition of initial presentation, based on imaging or abnormal liver function tests, likely lags behind symptom onset or first clinical contact. Finally, we did not account for patients undergoing multiple EUS procedures, which may reflect more complex diagnostic courses and could influence estimates of diagnostic timing and downstream outcomes.

EUS is a critical tool for diagnosing and staging pancreatic cancer. Although the American Society for Gastrointestinal Endoscopy and American College of Gastroenterology have recently published a set of proposed quality metrics for EUS performance (35), time to tissue sampling of pancreatic lesions was not among the listed criteria. Our findings highlight the importance of diagnostic timeliness as a potentially meaningful quality metric. Future guidelines that define acceptable delay intervals and incorporate timeliness into oncologic care pathways may help optimize the diagnosis and treatment of pancreatic cancer.

## CONFLICTS OF INTEREST

**Guarantor of the article:** Jingchuan Guo, PhD.

**Specific author contributions:** R.M.R.: methodology, data curation, data analysis, visualization, writing—original draft, writing—review and editing. W.H.: methodology, data curation, data analysis. G.B.: conceptualization, methodology, writing—original draft, writing—review and editing. J.F.: conceptualization, methodology, supervision, writing—review and editing. J.G.: conceptualization, methodology, project supervision, project administration, funding acquisition, writing—review and editing. A.N.: conceptualization, methodology, project supervision, project administration, writing—original draft, writing—review and editing.

**Financial support:** None to report.

**Potential competing interests:** None to report.

**Study Highlights**

**WHAT IS KNOWN**

✓ Pancreatic cancer has poor survival; early diagnosis is critical.
✓ Care delays in pancreatic cancer occur, but definitions and impacts remain undefined.
✓ Disparities exist across the pancreatic cancer diagnostic and treatment continuum.

**WHAT IS NEW HERE**

✓ First national study evaluating time-to-EUS in older adults with pancreatic cancer.
✓ Approximately 21% experienced >30-day delay to EUS from time of onset of symptoms.
✓ Black patients had significantly higher odds of delayed EUS.
✓ > 30-day delay in EUS is associated with a lower likelihood of surgical resection.

## Supplementary Material

## ABBREVIATIONS:

ACG: American College of Gastroenterology
ADI: Area Deprivation Index
aOR: Adjusted Odds Ratio
ASGE: American Society for Gastrointestinal Endoscopy
CCI: Charlson Comorbidity Index
CI: Confidence Interval
CPT: Current Procedural Terminology
ERCP: Endoscopic Retrograde Cholangiopancreatography
EUS: Endoscopic Ultrasound
FNB: Fine Needle Biopsy
HR: Hazard Ratio
ICD-10: International Classification of Diseases
Tenth Revision: ICD-9
International Classification of Diseases: Ninth Revision
IQR: Interquartile Range
LFTs: Liver Function Tests
SD: Standard Deviation
STROBE: Strengthening the Reporting of Observational Studies in Epidemiology

## References

[1] Wood LD Canto MI Jaffee EM Pancreatic cancer: Pathogenesis, screening, diagnosis, and treatment. Gastroenterology 2022;163(2):386–402.e1. 35398344 10.1053/j.gastro.2022.03.056 PMC9516440

[2] Kolbeinsson HM Chandana S Wright GP Pancreatic cancer: A review of current treatment and novel therapies. J Invest Surg 2023;36(1):2129884. 36191926 10.1080/08941939.2022.2129884

[3] Macedo FI Ryon E Maithel SK Survival outcomes associated with clinical and pathological response following neoadjuvant FOLFIRINOX or Gemcitabine/nab-paclitaxel chemotherapy in resected pancreatic cancer. Ann Surg 2019;270(3):400–13. 31283563 10.1097/SLA.0000000000003468 PMC9634701

[4] Rustgi SD Zylberberg HM Amin S Use of endoscopic ultrasound for pancreatic cancer from 2000 to 2016. Endosc Int Open 2022;10(1):E19–E29. 35047331 10.1055/a-1608-0856 PMC8759943

[5] Gonzalo-Marin J Vila JJ Perez-Miranda M. Role of endoscopic ultrasound in the diagnosis of pancreatic cancer. World J Gastrointest Oncol 2014;6(9):360–8. 25232461 10.4251/wjgo.v6.i9.360 PMC4163734

[6] Salom F Prat F. Current role of endoscopic ultrasound in the diagnosis and management of pancreatic cancer. World J Gastrointest Endosc 2022;14(1):35–48. 35116098 10.4253/wjge.v14.i1.35 PMC8788172

[7] ASGE Standards of Practice Committee, Eloubeidi MA Decker GA The role of endoscopy in the evaluation and management of patients with solid pancreatic neoplasia. Gastrointest Endosc 2016;83(1):17–28. 26706297 10.1016/j.gie.2015.09.009

[8] Zakaria A Al-Share B Klapman JB The role of endoscopic ultrasonography in the diagnosis and staging of pancreatic cancer. Cancers (Basel) 2022;14(6):1373. 35326524 10.3390/cancers14061373 PMC8946253

[9] Ngamruengphong S Li F Zhou Y EUS and survival in patients with pancreatic cancer: A population-based study. Gastrointest Endosc 2010;72(1):78–83.e832. 20620274 10.1016/j.gie.2010.01.072

[10] Jooste V Dejardin O Bouvier V Pancreatic cancer: Wait times from presentation to treatment and survival in a population-based study. Int J Cancer 2016;139(5):1073–80. 27130333 10.1002/ijc.30166

[11] Croome KP Chudzinski R Hanto DW. Increasing time delay from presentation until surgical referral for hepatobiliary malignancies. HPB (Oxford) 2010;12(9):644–8. 20961373 10.1111/j.1477-2574.2010.00217.x PMC2999792

[12] McLean SR Karsanji D Wilson J The effect of wait times on oncological outcomes from periampullary adenocarcinomas. J Surg Oncol 2013;107(8):853–8. 23625192 10.1002/jso.23338

[13] Bilimoria KY Ko CY Tomlinson JS Wait times for cancer surgery in the United States: Trends and predictors of delays. Ann Surg 2011;253(4):779–85. 21475020 10.1097/SLA.0b013e318211cc0f

[14] Allan AC Gamaldo AA Wright RS Differential associations between the area deprivation index and measures of physical health for older Black adults. J Gerontol B Psychol Sci Soc Sci 2023;78(2):253–63. 36161476 10.1093/geronb/gbac149 PMC9938923

[15] Vintimilla R Seyedahmadi A Hall J Association of area deprivation index and hypertension, diabetes, dyslipidemia, and obesity: A cross-sectional study of the HABS-HD cohort. Gerontol Geriatr Med 2023;9:23337214231182240. 37361029 10.1177/23337214231182240 PMC10286155

[16] Charlson ME Pompei P Ales KL A new method of classifying prognostic comorbidity in longitudinal studies: Development and validation. J Chronic Dis 1987;40(5):373-83. 3558716 10.1016/0021-9681(87)90171-8

[17] Roffman CE Buchanan J Allison GT. Charlson Comorbidities Index. J Physiother 2016;62(3):171. 27298055 10.1016/j.jphys.2016.05.008

[18] Silva-Santisteban A Hernandez Woodbine MJ Noriega MA Disparities in race, ethnicity, sex, and age inclusion in pancreatic cancer screening studies: A systematic review and meta-analysis. Gastrointest Endosc 2024;100(1):1–16.e20. 38432492 10.1016/j.gie.2024.02.014

[19] Regan J Zahnd W Kistner B Rural disparities in esophageal cancer outcomes based on distance from endoscopic ultrasound (EUS)-trained gastroenterologists. Gastroenterology 2015;148(4):S–1157.

[20] Fallon J Standring O Vithlani N Minorities face delays to pancreatic cancer treatment regardless of diagnosis setting. Ann Surg Oncol 2024;31(8):4986–96. 38789617 10.1245/s10434-024-15352-3 PMC11236843

[21] Murphy MM Simons JP Ng SC Racial differences in cancer specialist consultation, treatment, and outcomes for locoregional pancreatic adenocarcinoma. Ann Surg Oncol 2009;16(11):2968–77. 19669839 10.1245/s10434-009-0656-5

[22] Tavakkoli A Singal AG Waljee AK Racial disparities and trends in pancreatic cancer incidence and mortality in the United States. Clin Gastroenterol Hepatol 2020;18(1):171–8.e10. 31202981 10.1016/j.cgh.2019.05.059

[23] Noel M Fiscella K. Disparities in pancreatic cancer treatment and outcomes. Health Equity 2019;3(1):532–40. 31663065 10.1089/heq.2019.0057 PMC6818479

[24] Bohan RP Riner AN Herremans KM Impact of biopsy attempts, race, and access on time to initiation of treatment for pancreatic cancer. J Gastrointest Surg 2023;27(11):2474–83. 37740146 10.1007/s11605-022-05531-6 PMC11220574

[25] Deshwar AB Sugar E Torto D Diagnostic intervals and pancreatic ductal adenocarcinoma (PDAC) resectability: A single-center retrospective analysis. Ann Pancreat Cancer 2018;1:13. 29683142 10.21037/apc.2018.02.01 PMC5909699

[26] Yu J Blackford AL Dal Molin M Time to progression of pancreatic ductal adenocarcinoma from low-to-high tumour stages. Gut 2015;64(11):1783–9. 25636698 10.1136/gutjnl-2014-308653 PMC4520782

[27] Ahn SJ Choi SJ Kim HS. Time to progression of pancreatic cancer: Evaluation with multi-detector computed tomography. J Gastrointest Cancer 2017;48(2):164–9. 27699624 10.1007/s12029-016-9876-7

[28] de Leon Pisani R Archibugi L Lazzano P Diagnostic delay at diagnosis and time-to-treatment influence overall survival of pancreatic cancer patients. Dig Liver Dis 2025;57(6):1308–14. 40175166 10.1016/j.dld.2025.03.009

[29] Stornello C Archibugi L Stigliano S Diagnostic delay does not influence survival of pancreatic cancer patients. United Eur Gastroenterol J 2020;8(1):81–90. 10.1177/2050640619879004 PMC7006002 32213057

[30] Walter FM Mills K Mendonça SC Symptoms and patient factors associated with diagnostic intervals for pancreatic cancer (SYMPTOM pancreatic study): A prospective cohort study. Gastroenterol Hepatol 2016;1(4):298-306. 10.1016/S2468-1253(16)30079-6 PMC6358142 28404200

[31] Gobbi PG Bergonzi M Comelli M The prognostic role of time to diagnosis and presenting symptoms in patients with pancreatic cancer. Cancer Epidemiol 2013;37(2):186–90. 23369450 10.1016/j.canep.2012.12.002

[32] Raptis DA Fessas C Belasyse-Smith P Clinical presentation and waiting time targets do not affect prognosis in patients with pancreatic cancer. Surgeon 2010;8(5):239–46. 20709279 10.1016/j.surge.2010.03.001

[33] Lukács G Kovács Á Csanádi M Benefits of timely care in pancreatic cancer: A systematic review to navigate through the contradictory evidence. Cancer Manag Res 2019;11:9849–61. 31819622 10.2147/CMAR.S221427 PMC6875504

[34] Sanjeevi S Ivanics T Lundell L Impact of delay between imaging and treatment in patients with potentially curable pancreatic cancer. Br J Surg 2016;103(3):267–75. 26572509 10.1002/bjs.10046

[35] Mishra G Lennon AM Pausawasdi N Quality indicators for EUS. Gastrointest Endosc 2025;101(5):928–49.e1. 40266165 10.1016/j.gie.2025.02.025

