# Supplementary material for: Evaluation of Endoscopic Ultrasound Delays in the Diagnosis of Pancreatic Cancer in Older Adults in the United States
Source: Clin Transl Gastroenterol. 2026 Jan 30;17(4):e00986. doi: 10.14309/ctg.0000000000000986 (PMC13102446; doi:10.14309/ctg.0000000000000986)
Supplement: Supplementary file 1 [file ct9-17-e00986-s001.docx]

**Title:** Evaluation of Endoscopic Ultrasound Delays in the Diagnosis of Pancreatic Cancer in Older Adults in the United States

**Content:**

**Table S1.** Multivariable Logistic Regression Results of Factors Associated with Diagnostic Delay (>8 days)

**Table S2.** Multivariable Cox Proportional Hazards Model of Time to Pancreatic Surgery Using >8 Day Delay Threshold

**Table S3.** Multivariable Cox Proportional Hazards Model of All-Cause Mortality Using >8 Day Delay Threshold

**Table S4.** Multivariable Logistic Regression Results of Factors Associated with Diagnostic Delay (>60 days)

**Table S5.** Multivariable Cox Proportional Hazards Model of Time to Pancreatic Surgery Using >60 Day Delay Threshold

**Table S6.** Multivariable Cox Proportional Hazards Model of All-Cause Mortality Using >60 Day Delay Threshold

| **Table 1. Multivariable Logistic Regression Results of Factors Associated with Diagnostic Delay (>8 days)** | | | |
| --- | --- | --- | --- |
| **Variable** | **aOR** | **95% CI** | **p-value** |
| **Area Deprivation Index** | 1.01 | 1.00–1.01 | 0.006 |
| **Charlson Comorbidity Index** | 0.94 | 0.91–0.98 | 0.001 |
| **Age (ref: <75 years)** |  |  |  |
| ≥75 years | 0.87 | 0.73–1.04 | 0.125 |
| **Sex (ref: Female)** |  |  |  |
| Male | 1.00 | 0.81–1.24 | 0.986 |
| **Race/Ethnicity (ref: White)** |  |  |  |
| Black | 1.17 | 0.80–1.70 | 0.417 |
| Hispanic | 1.00 | 0.63–1.60 | 0.989 |
| Other | 1.08 | 0.73–1.58 | 0.704 |
| **Region (ref: Southeast)** |  |  |  |
| West | 1.21 | 0.93–1.58 | 0.164 |
| Midwest | 0.95 | 0.77–1.18 | 0.660 |
| Northeast | 1.08 | 0.87–1.35 | 0.473 |
| Southwest | 0.97 | 0.71–1.32 | 0.829 |
| **Residence Location (ref: Rural)** |  |  |  |
| Urban | 0.94 | 0.74–1.19 | 0.603 |
| * Additional covariates were adjusted in the model including: acute myocardial infarction, Alzheimer's disease, atrial fibrillation, cataract, chronic kidney disease, chronic obstructive pulmonary disease, congestive heart failure, diabetes, glaucoma, ischemic heart disease, depression, osteoporosis, rheumatoid arthritis and/or osteoarthritis, stroke and/or transient ischemic attack, anemia, asthma, hyperlipidemia, hyperparathyroidism, hypertension, hypothyroidism, alcohol use disorder, and obesity. | | | |

| **Table 2. Multivariable Cox Proportional Hazards Model of Time to Pancreatic Surgery** | | | |
| --- | --- | --- | --- |
| **Variable** | **HR** | **95% CI** | **p-value** |
| **Time-to-EUS (ref: ≤8 days)** |  |  |  |
| Long delay (>8 days) | 0.86 | 0.74–1.00 | 0.043 |
| **Area Deprivation Index** | 1.00 | 0.99–1.00 | 0.106 |
| **Charlson Comorbidity Index** | 0.95 | 0.91–0.99 | 0.020 |
| **Age (ref: <75 years)** |  |  |  |
| ≥75 years | 1.08 | 0.91–1.29 | 0.356 |
| **Sex (ref: Female)** |  |  |  |
| Male | 0.89 | 0.73–1.08 | 0.234 |
| **Race/Ethnicity (ref: White)** |  |  |  |
| Black | 0.68 | 0.44–1.06 | 0.090 |
| Hispanic | 1.36 | 0.90–2.05 | 0.141 |
| Other | 1.00 | 0.71–1.39 | 0.979 |
| **Region (ref: Southeast)** |  |  |  |
| West | 0.90 | 0.70–1.16 | 0.410 |
| Midwest | 0.87 | 0.71–1.07 | 0.179 |
| Northeast | 0.79 | 0.64–0.98 | 0.030 |
| Southwest | 0.82 | 0.59–1.12 | 0.210 |
| **Residence Location (ref: Rural)** |  |  |  |
| Urban | 0.84 | 0.67–1.07 | 0.155 |
| * Additional covariates were adjusted in the model including: acute myocardial infarction, Alzheimer's disease, atrial fibrillation, cataract, chronic kidney disease, chronic obstructive pulmonary disease, congestive heart failure, diabetes, glaucoma, ischemic heart disease, depression, osteoporosis, rheumatoid arthritis and/or osteoarthritis, stroke and/or transient ischemic attack, anemia, asthma, hyperlipidemia, hyperparathyroidism, hypertension, hypothyroidism, alcohol use disorder, and obesity. | | | |

| **Table 3. Multivariable Cox Proportional Hazards Model of Time to All- cause Mortality** | | | |
| --- | --- | --- | --- |
| **Variable** | **HR** | **95% CI** | **p-value** |
| **Time-to-EUS (ref: ≤8 days)** |  |  |  |
| Long delay (>8 days) | 0.82 | 0.74–0.91 | 0.000 |
| **Area Deprivation Index** | 1.00 | 1.00–1.01 | 0.193 |
| **Charlson Comorbidity Index** | 1.04 | 1.02–1.07 | 0.000 |
| **Age (ref: <75 years)** |  |  |  |
| ≥75 years | 1.44 | 1.27–1.82 | 0.000 |
| **Sex (ref: Female)** |  |  |  |
| Male | 1.00 | 0.87–1.15 | 0.977 |
| **Race/Ethnicity (ref: White)** |  |  |  |
| Black | 1.06 | 0.83–1.35 | 0.642 |
| Hispanic | 0.94 | 0.68–1.31 | 0.729 |
| Other | 0.78 | 0.59–1.04 | 0.088 |
| **Region (ref: Southeast)** |  |  |  |
| West | 1.05 | 0.87–1.26 | 0.625 |
| Midwest | 1.04 | 0.91–1.19 | 0.585 |
| Northeast | 1.05 | 0.91–1.22 | 0.497 |
| Southwest | 0.98 | 0.80–1.22 | 0.882 |
| **Residence Location (ref: Rural)** |  |  |  |
| Urban | 0.84 | 0.72–0.97 | 0.022 |
| * Additional covariates were adjusted in the model including: acute myocardial infarction, Alzheimer's disease, atrial fibrillation, cataract, chronic kidney disease, chronic obstructive pulmonary disease, congestive heart failure, diabetes, glaucoma, ischemic heart disease, depression, osteoporosis, rheumatoid arthritis and/or osteoarthritis, stroke and/or transient ischemic attack, anemia, asthma, hyperlipidemia, hyperparathyroidism, hypertension, hypothyroidism, alcohol use disorder, and obesity. | | | |

| **Table 4.** **Multivariable Logistic Regression Results of Factors Associated with Diagnostic Delay (>60 days)** | | | |
| --- | --- | --- | --- |
| **Variable** | **aOR** | **95% CI** | **p-value** |
| **Area Deprivation Index** | 1.00 | 0.99–1.01 | 0.959 |
| **Charlson Comorbidity Index** | 0.96 | 0.90–1.01 | 0.139 |
| **Sex (ref: Female)** |  |  |  |
| Male | 1.08 | 0.78–1.50 | 0.647 |
| **Age (ref: <75 years)** |  |  |  |
| ≥75 years | 0.72 | 0.54-0.95 | 0.020 |
| **Race/Ethnicity (ref: White)** |  |  |  |
| Black | 1.12 | 0.64–1.96 | 0.692 |
| Hispanic | 0.90 | 0.43–1.92 | 0.795 |
| Other | 1.30 | 0.75–2.23 | 0.348 |
| **Region (ref: Southeast)** |  |  |  |
| West | 1.26 | 0.84–1.88 | 0.270 |
| Midwest | 0.91 | 0.64–1.27 | 0.540 |
| Northeast | 1.18 | 0.84–1.66 | 0.332 |
| Southwest | 0.99 | 0.60–1.62 | 0.968 |
| **Residence Location (ref: Rural)** |  |  |  |
| Urban | 1.33 | 0.90–1.97 | 0.151 |
| * Additional covariates were adjusted in the model including: acute myocardial infarction, Alzheimer's disease, atrial fibrillation, cataract, chronic kidney disease, chronic obstructive pulmonary disease, congestive heart failure, diabetes, glaucoma, ischemic heart disease, depression, osteoporosis, rheumatoid arthritis and/or osteoarthritis, stroke and/or transient ischemic attack, anemia, asthma, hyperlipidemia, hyperparathyroidism, hypertension, hypothyroidism, alcohol use disorder, and obesity. | | | |

| **Table 5. Multivariable Cox Proportional Hazards Model of Time to Pancreatic Surgery** | | | |
| --- | --- | --- | --- |
| **Variable** | **HR** | **95% CI** | **p-value** |
| **Area Deprivation Index** | 1.00 | 0.99–1.00 | 0.090 |
| **Charlson Comorbidity Index** | 0.95 | 0.91–0.99 | 0.019 |
| **Time-to-EUS (ref: ≤60 days)** |  |  |  |
| Long delay (>60 days) | 0.73 | 0.57–0.92 | 0.009 |
| **Age (ref: <75 years)** |  |  |  |
| ≥75 years | 1.09 | 0.92–1.29 | 0.344 |
| **Sex (ref: Female)** |  |  |  |
| Male | 0.88 | 0.72–1.07 | 0.201 |
| **Race/Ethnicity (ref: White)** |  |  |  |
| Black | 0.68 | 0.44–1.05 | 0.081 |
| Hispanic | 1.32 | 0.88–1.99 | 0.184 |
| Other | 0.98 | 0.70–1.37 | 0.918 |
| **Region (ref: Southeast)** |  |  |  |
| West | 0.90 | 0.70–1.16 | 0.414 |
| Midwest | 0.87 | 0.71–1.07 | 0.198 |
| Northeast | 0.79 | 0.64–0.98 | 0.028 |
| Southwest | 0.81 | 0.59–1.12 | 0.207 |
| **Residence Location (ref: Rural)** |  |  |  |
| Urban | 0.85 | 0.67–1.08 | 0.178 |
| * Additional covariates were adjusted in the model including: acute myocardial infarction, Alzheimer's disease, atrial fibrillation, cataract, chronic kidney disease, chronic obstructive pulmonary disease, congestive heart failure, diabetes, glaucoma, ischemic heart disease, depression, osteoporosis, rheumatoid arthritis and/or osteoarthritis, stroke and/or transient ischemic attack, anemia, asthma, hyperlipidemia, hyperparathyroidism, hypertension, hypothyroidism, alcohol use disorder, and obesity. | | | |

| **Table 6. Multivariable Cox Proportional Hazards Model of Time to All-cause Mortality** | | | |
| --- | --- | --- | --- |
| **Variable** | **HR** | **95% CI** | **p-value** |
| **Area Deprivation Index** | 1.00 | 1.00–1.00 | 0.233 |
| **Charlson Comorbidity Index** | 1.04 | 1.02–1.07 | 0.000 |
|  |  |  |  |
|  |  |  |  |
| **Time-to-EUS (ref: ≤60 days)** |  |  |  |
| Long delay (>60 days) | 0.62 | 0.51–0.74 | <0.001 |
| **Age (ref: <75 years)** |  |  |  |
| ≥75 years | 1.43 | 1.27–1.61 | <0.001 |
| **Sex (ref: Female)** |  |  |  |
| Male | 1.00 | 0.87–1.15 | 0.965 |
| **Race/Ethnicity (ref: White)** |  |  |  |
| Black | 1.06 | 0.83–1.35 | 0.642 |
| Hispanic | 0.94 | 0.67–1.31 | 0.711 |
| Other | 0.78 | 0.59–1.04 | 0.089 |
| **Region (ref: Southeast)** |  |  |  |
| West | 1.04 | 0.87–1.25 | 0.666 |
| Midwest | 1.04 | 0.90–1.20 | 0.587 |
| Northeast | 1.05 | 0.91–1.22 | 0.521 |
| Southwest | 0.98 | 0.79–1.21 | 0.854 |
| **Residence Location (ref: Rural)** |  |  |  |
| Urban | 0.85 | 0.73–0.99 | 0.039 |
| * Additional covariates were adjusted in the model including: acute myocardial infarction, Alzheimer's disease, atrial fibrillation, cataract, chronic kidney disease, chronic obstructive pulmonary disease, congestive heart failure, diabetes, glaucoma, ischemic heart disease, depression, osteoporosis, rheumatoid arthritis and/or osteoarthritis, stroke and/or transient ischemic attack, anemia, asthma, hyperlipidemia, hyperparathyroidism, hypertension, hypothyroidism, alcohol use disorder, and obesity. | | | |
